# India Policy Insights: A geospatial and temporal data science and visualization platform and architecture

**DOI:** 10.1016/j.softx.2025.102149

**Published:** 2025-05

**Authors:** Devika Jain, Joaquin Kachinovsky, Gonzalo Rodriguez, Junyi Chen, Rockli Kim, S V Subramanian

**Affiliations:** a Center for Geographic Analysis, Harvard University, 1737 Cambridge Street, Cambridge, MA 02138, USA; b Onetree, 425 Urban Street, Kirkland, WA 98033, USA; c McCormick School of Engineering, Northwestern University, Evanston, IL, USA; d Division of Health Policy and Management, College of Health Science, Korea University, 145 Anam-ro, Seongbuk-gu, Seoul 02841, South Korea; e Interdisciplinary Program in Precision Public Health, Department of Public Health Sciences, Graduate School of Korea University, 145 Anam-ro, Seongbuk-gu, Seoul 02841, South Korea; f Harvard Center for Population and Development Studies, 9 Bow Street, Cambridge, MA 02138, USA; g Department of Social and Behavioral Sciences, Harvard T. H. Chan School of Public Health, 677 Huntington Avenue, Boston, MA 02115, USA

**Keywords:** Data insights, Docker, Azure, Databases, .NET/React, ETL

## Abstract

The Geographic Insights Lab at Harvard University developed India Policy Insights (IPI), a spatio-temporal visualization platform for policymakers. IPI provides insights from 122 indicators across population, health, and socioeconomic metrics spanning 720 districts, 543 parliamentary constituencies, and 600,000 villages in India. Its applications include breastfeeding campaigns,policy development, and government reporting. It is fully deployed on Microsoft Azure using Docker, which ensures scalability and reproducibility. Built on an open-source stack with React,.NET, and PostGIS, it processes, stores, visualizes, and queries geospatial big data. This paper highlights IPI's architecture and methodologies for tackling public policy challenges.

**Metadata**

**Table:** 

**Nr**	**Code metadata description**	***Metadata***
C1	Current code version	V1
C2	Permanent link to code/repository used for this code version	https://github.com/onetree-com/india-policy-insights-frontend https://github.com/onetree-com/india-policy-insights-backend
C3	Permanent link to reproducible capsule	https://github.com/onetree-com/india-policy-insights-backend/blob/main/src/docker-compose.yml
C4	Legal code license	MIT License The MIT License is a permissive open-source license that allows users to freely use, copy, modify, and distribute the software for personal or commercial purposes. The key conditions are: **Attribution:** Users must include the original copyright notice and license text in any copies or substantial portions of the software. **No Warranty:** The software is provided "as is," without any warranties, making the authors not liable for any issues arising from its use. This simplicity and flexibility make the MIT License widely adopted and suitable for various projects.
C5	Code versioning system used	Git
C6	Software code languages, tools and services used	C#, .NET Core 3.1, ReactJS, PostgreSQL, TileServerGL
C7	Compilation requirements, operating environments and dependencies	Windows or Linux operating system with Docker Desktop, Node.js and GIT. In the case of Windows it is recommended to use WSL 2 (Windows Subsystem for Linux) for cross compatibility of the images between operating systems.
C8	If available, link to developer documentation/manual	https://github.com/onetree-com/india-policy-insights-backend/blob/main/README.md and https://github.com/onetree-com/india-policy-insights-backend/blob/main/setup.md
C9	Support email for questions	svsubram@hsph.harvard.edu

## 1 Motivation and significance

Effective public policy increasingly relies on geospatial data visualization to offer critical insights into regional disparities and inform localized decision-making. As policy challenges become more complex and require targeted interventions, geo-visualization tools have emerged as indispensable assets for identifying patterns and disparities across geographic scales [1]. GIS usage in public health research has grown significantly, especially post-COVID-19, highlighting its effectiveness in shaping public health policy [2]. Unlike traditional Geographic Information Systems (GIS), which often demand specialized expertise, modern platforms incorporate interactive, user-friendly features. These advancements democratize access to geospatial analytics, empowering a wide range of users—from policymakers to analysts—to interpret and act on complex spatial data effectively [3].

Interactive mapping tools, such as React, .Net, D3.js, and other advanced visualization frameworks, facilitate multi-scale analysis, uncovering nuanced trends in socio-economic and health data. This capability enables policymakers to address local needs with precision, bridging the gap between raw data and actionable insights [1]. By visualizing public policy data to highlight regional disparities, these tools support evidence-based, location-specific policies essential for effective governance. Additionally, geo-visualization enhances transparency and accountability through alignment with open data principles, enabling public and government users to explore socio-economic trends and health outcomes interactively [4].

Technological advancements, including frameworks like React.js and PostgreSQL, play a pivotal role in ensuring the scalability, real-time processing, and accessibility of these tools, particularly when handling large, complex datasets [5]. By incorporating user-centred design principles, modern geospatial platforms cater to diverse expertise levels, offering intuitive interfaces and customized visualizations to facilitate informed decision-making [6].

Despite these advancements, a significant gap persists in the availability of high-quality datasets and tools for population, health, and socio-economic metrics, particularly at granular administrative levels [7]. Most mapping systems are extremely constrained when it comes to representing millions of features on a map unless the visualization has been previously generated [8]. Additionally, handling big data comes with key challenges, including the vast volume of data, ensuring data quality and integration, privacy and security concerns, real-time data processing, and the incorporation of predictive insights into clinical practice [9]. Addressing this gap is critical for fostering data-driven governance and supporting sustainable development goals. To meet these needs, the Geographic Insights Lab at Harvard University developed the India Policy Insights (IPI) platform—a spatiotemporal visualization tool designed to make geospatial data more accessible and actionable for policymakers.

India Policy India Insights Dataset consists of 122 indicators covering population, health, and socio-economic metrics across 720 districts, 543 parliamentary constituencies (PCs), and 600,000 villages in India [10] from 2016 to 2021. Using 2016 and 2021 National Family Health Surveys [11,12] and other government data, we estimate prevalence ( %), progress points, and headcounts (for 2021) for 122 indicators across 720 districts and 543 PCs. The dataset is updated every three to five years. Several quality checks, validation processes, and sensitivity tests were implemented to ensure dataset accuracy and reliability. Validation checks were performed at multiple stages, both before and after precision-weighted estimates. Weighted prevalence estimates were cross-referenced with figures from the NFHS India Report, with most indicators showing no or negligible differences. However, a few indicators could not be verified due to incompatibility in reference samples. Descriptive statistics (mean, median, IQR, histograms) were used to evaluate outcome distributions, compared against untreated figures. District and PC-level denominators were validated against 2011 Census data for precise headcount estimations. Final estimates underwent a rigorous quality review. The IPI open-source dataset can be found on Harvard Dataverse [10].

The IPI Data Explorer platform provides an interactive platform to visualize the prevalence and progress-based rankings of these geographies. This comprehensive tool supports applications ranging from breastfeeding campaigns to officer training and government report generation. Hosted on Microsoft Azure, with deployment powered by Docker, IPI ensures scalability and reproducibility. Built on an open-source technology stack, including React, .NET, and PostGIS, the platform integrates advanced methodologies for processing, enriching, and visualizing geospatial big data. IPI also aligns well with the FAIR software development and data-sharing principles, specifically:

• **Findable (F):** A persistent and openly available repository for both data and software.
• **Accessible (A):** A fully open-source software stack ensuring transparency and flexibility.
• **Interoperable (I):** Cross-platform compatibility using a Docker-based stack for seamless integration.
• **Reusable (R):** Full customization of both front-end and back-end to meet diverse user needs. Also, scalability and high-performance for geospatial big data.

This paper highlights the IPI platform's methodologies and architecture, demonstrating its effectiveness in addressing pressing public policy challenges. By bridging the gap between data accessibility and actionable insights, IPI represents a significant step forward in leveraging geospatial technologies to enhance evidence-based policymaking and sustainable development.

## 2 Software description

The India Policy Insights (IPI) platform is a spatio-temporal platform designed to derive actionable policy insights from population, health, and socioeconomic indicators across multiple geographic levels.

### 2.1 Software architecture

**Fig. 1:**
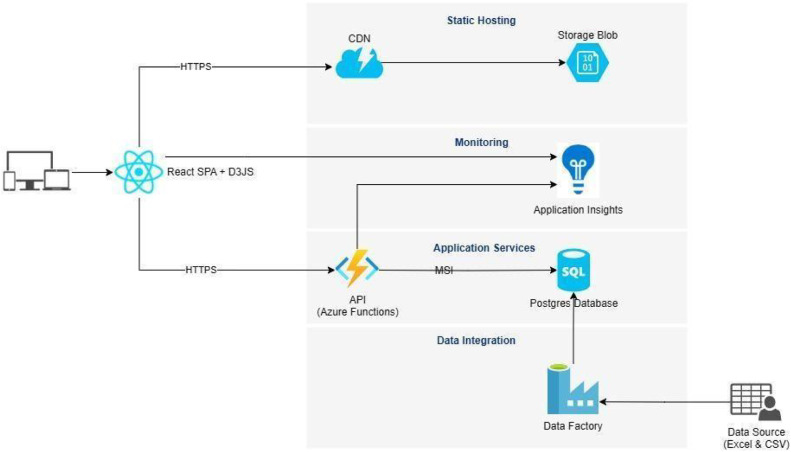
An overview of the software architecture of India Policy Insights (IPI).

The IPI system architecture integrates advanced components for scalability, performance, reusability, and easy maintenance (Fig. 1). The three key components are described below.

#### 2.1.1 Backend

The backend of the system is developed using ASP.NET Core [13], a cross-platform, open-source framework by Microsoft. ASP.NET Core, an evolution of the widely used ASP.NET framework, offers significant improvements in performance and flexibility, making it an ideal choice for building scalable, high-performance web applications. The backend is responsible for executing core business logic, managing API endpoints, and integrating with third-party services for authentication and data processing.

The deployment process is simplified by the ability to directly import the Azure Functions [14] publish profile into Visual Studio [15], streamlining the deployment to the Azure environment. Additionally, the backend is designed to scale efficiently, utilizing Azure's container services [16] to manage load balancing and handle traffic increases seamlessly. This scalable architecture ensures robust performance even during periods of high demand, providing an optimal user experience.

#### 2.1.2 Frontend

The frontend of our platform is built using React.js [17], a widely used open-source JavaScript library known for its component-based architecture, which enables the creation of dynamic and high-quality user interfaces. React is paired with D3.js [18], another open-source tool specifically designed for data visualization. D3.js is lightweight, efficient in handling large datasets, and highly flexible, allowing the development of complex visualizations that significantly enhance the user experience. For deployment, the React application is built using npm commands, with environment-specific variables configured in package.json. After the build process, the content from the build folder is uploaded to the $web folder of Azure Blob Storage [19]. To ensure users access the most up-to-date version of the application, we performed a CDN [20] purge after each update.

#### 2.1.3 Database

We use PostgreSQL [21], a robust database system optimized for large datasets with features like table partitioning and column store indexing. Data ingestion and updates are automated using Azure Data Factory [22], which processes uploaded .csv files via Data Flow before inserting them into indexed, read-optimized PostgreSQL tables for efficient management and timely updates.

Geographic shapefiles were transformed into web-compatible vector tiles using QGIS [23] and Mapbox Tippecanoe [24], producing mbtiles files served via TileServer [25] to support map functionality. The IPI database Entity-Relationship Model is available on GitHub [26].

### 2.2 Software deployment

India Policy Insights (IPI) is deployed on Microsoft Azure Cloud, which is a scalable and secure cloud platform. The deployment is containerized using Docker and is fully monitored using Azure Insights. More details about the deployment are described in sections below.

#### 2.2.1 Azure cloud

India Policy Insights (IPI) is deployed on Microsoft Azure Cloud [27] and utilizes Azure Functions [14], which are serverless compute services that enable developers to run event-triggered code without having to manage infrastructure. Key Azure components include:

• **app-ipi-linux**: Core compute resource on Azure Virtual Machines, ensuring reliable backend operations with average CPU usage of *14 %* and memory at *71 %*.
• **ipi-postgres-prod**: Primary database hosted on Azure Database for PostgreSQL supporting *351,091* successful connections with an average CPU usage of *10 %*, memory at *54 %*, and *68 %* storage utilization.
• **app-ipi-mysql**: Auxiliary database on Azure Database for MySQL [28] with robust query handling, *8 %* average CPU usage, and *21 %* memory utilization.
• **app-ipi-tiles**: Map tile service deployed via Azure App Service, leveraging containerized images for efficient geo-visualization.
• **ipi-api-prod**: Performance monitoring with Azure Application Insights, achieving a *131.9* ms average server response time and handling *1230* requests with minimal failures.
• **app-ipi-explorer**: User interface on Azure App Service [29], ensuring responsive interactions via custom and default domains.
• **ipi-wordpress**: Content management platform deployed on Azure App Service, accessible through default and custom domains.

#### 2.2.2 Docker orchestration

IPI's backend is fully deployed on a Docker-based [30] infrastructure within Azure. This ensures consistent, replicable environments by packaging applications with dependencies in isolated containers. It enables deployment on any Docker-compatible service, supporting scalability and resource efficiency. Security is managed at both container and platform levels, while Azure integration ensures seamless scaling and efficient resource management.

#### 2.2.3 Monitoring

Application Insights [31] service is employed for comprehensive monitoring of both the frontend and backend components. This service delivers real-time analytics on platform performance, enabling the identification of failures and performance bottlenecks. The dashboard supports detailed analysis of specific requests, including failure diagnostics. For instance, users can trace failed requests to view their content and dependencies, such as database calls, providing valuable insights for debugging and optimization. This modular and scalable architecture ensures the IPI platform delivers a seamless user experience while maintaining robustness and adaptability for future enhancements.

### 2.3 Software performance

We performed the following stress and load testing to test the system performance. It demonstrates the system's robustness and ability to maintain reliable performance under varying load conditions. The sections below describe the various test scenarios and details about which can be found on our GitHub [32].

#### 2.3.1 Development

We simulated *10* users making requests at *1-second* intervals to reflect usage during testing and development, allowing insights into basic performance and resource allocation. The average response time was *211.7* ms across *120* transactions with *zero* failed transactions. Table 1 below summarizes the results of the development scenario.

**Table 1:** Summary of Results: Development scenario.

Test threads (seconds - repetitions)	10 (1 - 1)
HTTP Requests	12
Test duration	6 s
Average time per transaction	211.75 ms
total transactions	120
failed transactions	0
correct transactions	120

#### 2.3.2 Production

We emulated a realistic, production-like environment, with *50* concurrent users performing requests, some of which included dynamic variables. This scenario assessed the backend's capacity to handle typical operational loads. The average response time was *149.7* ms across *600* transactions with *zero* failed transactions. Table 2 below summarizes the results for the production scenario.

**Table 2:** Summary of Results: Production scenario.

Test threads (seconds - repetitions)	50 (1 - 1)
HTTP Requests	12
Test duration	2 s
Average time per transaction	149.7 ms
total transactions	600
failed transactions	0
correct transactions	600

#### 2.3.3 Saturationn

We tested the backend under maximum load conditions by simulating *100* concurrent users, observing the point at which the system approaches its performance threshold. The average response time was *133.0* ms across *2400* transactions with *zero* failed transactions. Table 3 below summarizes the results for the saturation scenario.

**Table 3:** Summary of Results: Saturation scenario.

Test threads (seconds - repetitions)	100 (1 - 2)
HTTP Requests	12
Test duration without error	4 s
Test duration	4 s
Average time per transaction without error	133 ms
Average time per transaction	133 ms
total transactions	2400
failed transactions	0
correct transactions	2400

#### 2.3.4 Performance comparison

To assess the performance of India Policy Insights (IPI) against off-the-shelf geospatial visualization tools, we uploaded IPI data to these platforms and evaluated the key metrics, including data size, loading time, interactivity, and customization. Table 4 presents the results, including best-case scenarios and sample dashboards created using these platforms with IPI data.

**Table 4:** Performance Comparison of IPI with Other Visualization Software.

Platform	Data Size	Time to load	Interactivity	Customization	Best Use case	Dashboard
**India Policy Insights (IPI)**	500,000 polygons, 122 indicators	Real-time	High (dynamic filtering, comparison, ranking)	Fully customizable	Large-scale public policy & SDG analytics	https://tinyurl.com/msctr7xm
**Power BI**	1500 polygons (maximum)	Slow	Limited	Restricted design and geospatial display system	Business intelligence, basic geospatial reporting	https://tinyurl.com/mvakkrm3
**Tableau**	4000 polygons, 3 indicators	Very slow	Limited, lacks filters and indicator selection for big data	Minimal	Enterprise dashboarding with simple GIS overlays	https://tinyurl.com/52b8s2f4
**Mapbox (Power BI integration)**	30,000 points/polygons (maximum)	Slow	Limited	Restricted to MapBox's style/design	Custom web maps with limited scalability	https://tinyurl.com/3vpkayba
**Kepler.gl**	100,000 points (not polygons)	Fast for point data	Moderate	Restricted (one indicator at a time)	High-speed visualization of points data	https://tinyurl.com/ycxh6kta

As shown in Table 4, IPI outperforms Tableau, Power BI, and Mapbox in handling large geospatial datasets, supporting 500,000 polygons and 122 indicators. Power BI allows only 1500 polygons (except for U.S. counties) and has long load times for large datasets. Tableau struggles with slow performance and lacks efficient filtering for large datasets; for example, our Assembly Constituency dashboard with 3 indicators for 4000 polygons takes a long time to load. Mapbox (within Power BI) inherits these limitations and further restricts display to 30,000 points or polygons while locking users into its map styles. Kepler.gl offers fast visualization but is limited to point-based data, supporting only one indicator at a time—requiring separate sites for each, as in our Village Undernutrition analysis. In contrast, IPI ensures real-time rendering and analysis via optimized PostGIS queries on Azure, making it more efficient than the alternatives. With advanced filtering, comparison, and ranking, IPI provides a more interactive experience than traditional BI tools. Overall, IPI is the most effective solution for large-scale, multi-indicator geospatial analytics.

### 2.4 System functionalities

**Fig. 2:**
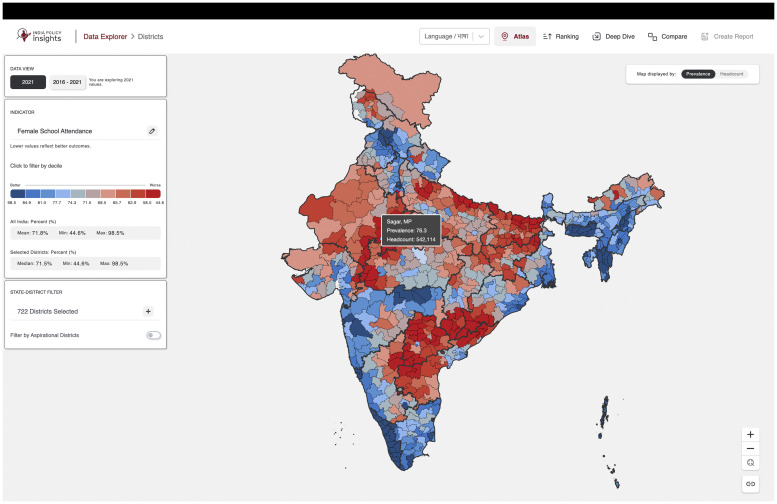
An overview of Atlas feature showing map of Female School Attendance for all 722 Districts. *Note: While there are 722 polygons, data is not available for 2 districts in Jammu and Kashmir, as the survey was not done there.

The IPI Data Explorer, as shown in Fig. 2, is a comprehensive geostatistical tool designed to support data-driven policy analysis and evaluation by visualizing health, education, and development indicators across India. Users can access various features, each of which is designed to provide a unique view of the data and facilitate an intuitive analysis of governmental policy impacts across different geographic divisions. The IPI data explorer is accessible here: https://tinyurl.com/msctr7xm [33]. A detailed user manual describing each of these functionalities can be found on our GitHub here: https://tinyurl.com/mtx83d3w [34]. An introduction video of the IPI data explorer can be found here: https://tinyurl.com/3xdy98vt. The system offers many functionalities that are described in the sections below.

#### 2.4.1 Atlas

Users can explore a single indicator across various geographic divisions—Districts, ACs, PCs, and Villages—selecting one division at a time from the landing page. The key features are described below.

• **Choropleth Mapping:** Color gradients (blue to red) highlight spatial patterns in indicator values.
• **Interactive Controls:** Zoom, pan, and reset tools provide both broad and detailed views.
• **Value Range Filters:** Display specific deciles or ranges to refine the map.
• **Shareable Links:** Generate short URLs to easily share current views.

A comprehensive training video on the Atlas feature can be found here: https://tinyurl.com/5n73533f.

#### 2.4.2 Deep dive

**Fig. 3:**
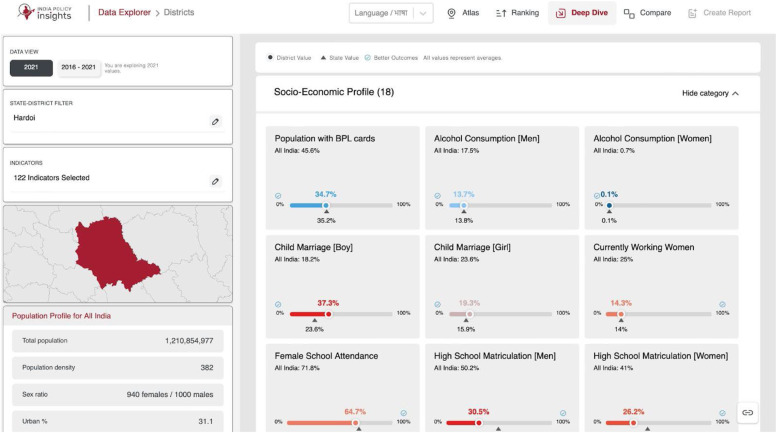
An overview of Deep Dive feature showing map of Socio-Economic Profile for Hardoi.

The Deep Dive feature, as shown in Fig. 3, provides detailed profiles of selected divisions, showing all indicators with state and national averages. The key features are described below.

• **Division Selection:** Choose divisions via menu or ranking list.
• **Demographics & Indicators:** Access demographic data (e.g., literacy) and indicator values.
• **Map Thumbnail:** View a static map of division boundaries.
• **Indicator Cards:** Indicator values appear as cards with percentage bars and comparisons.
• **Time Setting:** Compare current and historical data, with trends highlighted in Change Mode.

A comprehensive training video on the Deep feature can be found here: https://tinyurl.com/yc5y74xd.

#### 2.4.3 Compare

**Fig. 4:**
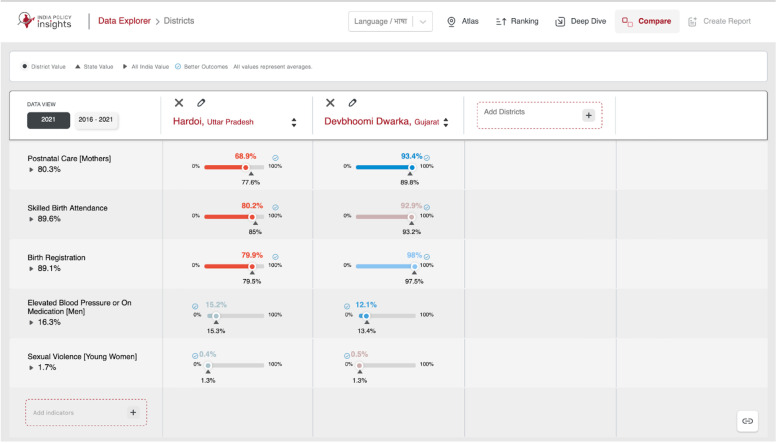
An overview of Compare feature showing comparison between districts of Hardoi and Dwarka.

The Compare feature, as shown in Fig. 4, allows side-by-side analysis of up to four divisions of the same type across multiple indicators. The key features are described below.

• **Selection:** Choose divisions and indicators with checkboxes and text search.
• **Comparison Matrix:** Divisions appear as columns and indicators as rows for structured comparisons.
• **Sorting & Filtering:** Sort by values or filter to focus on selected indicators.
• **Time Setting:** Compare historical data with Change Mode highlighting trends like "Improved" or "Worsened."

A comprehensive training video on the Compare feature video can be found here: https://tinyurl.com/yaxsvkuf.

#### 2.4.4 Ranking

**Fig. 5:**
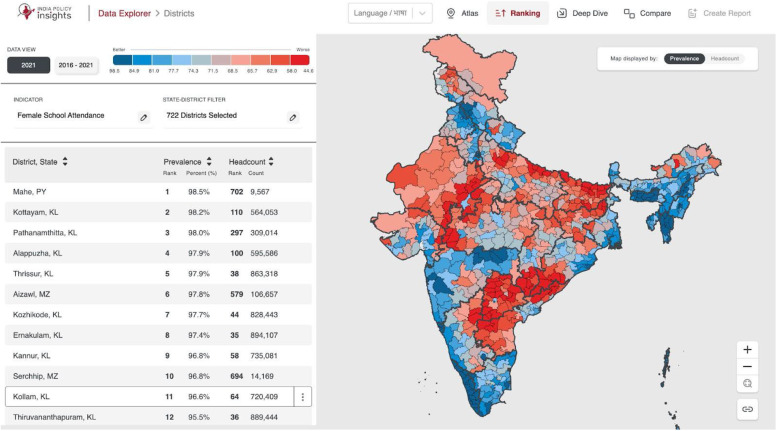
An overview of the Ranking feature showing districts ranked by Female School Attendance.

The Ranking feature, as shown in Fig. 5, ranks divisions by performance on a selected indicator. The key features are described below.

• **Rankings:** Sort divisions best-to-worst or vice versa by indicator values.
• **Filters:** Select indicators and filter by division type or aspirational districts.
• **Map & List Views:** Desktop shows a color-coded map; mobile displays a ranking list.
• **Time Comparison:** Compare current and historical rankings to track changes.

A comprehensive training video on the Ranking feature video can be found here: https://tinyurl.com/yc65nhzw.

#### 2.4.5 Reports*

*Note: The current version of Reports is illustrative and therefore not made functional in the actual dashboard. It is intended to conceptually highlight the importance of including such a feature in a policy dashboard.

**Fig. 6:**
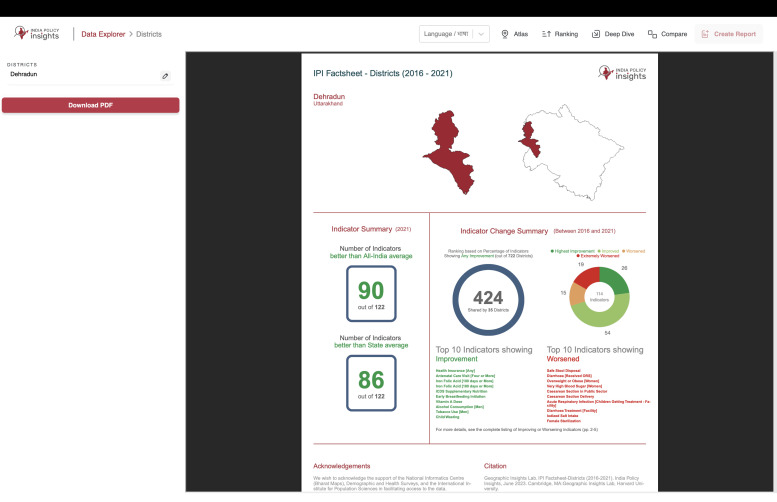
Overview of Report feature showing IPI Factsheet generated for districts.

The Report Generator, as shown in Fig. 6, creates downloadable PDF reports with detailed indicator data for selected divisions. The key features are described below.

• **Report Generation:** Users can preview and download customized reports.
• **Data Content:** Includes demographic details, indicator values, trends, and year-over-year comparisons.
• **Graphs & Maps:** Features bar graphs comparing state/national averages and village-level choropleth maps showing intra-district variation.

#### 2.4.6 Villages

The Village-Level Data feature is tailored for India's vast number of villages, optimizing display and functionality. The key features are described below.

• **District Selection:** Users must select a district to view villages, with one district displayed at a time.
• **Current Data Only:** Only current data is available; change tracking is not supported.
• **Filters & Highlights:** Filter villages by indicators or aspirational district status, and highlight specific villages for detailed indicator values.
• **Data Bars:** Display mean indicator values for India, the district, and filtered/highlighted villages.

## 3 Illustrative examples

India Policy Insights (IPI) offers a user-friendly interface for in-depth analysis and visualization of critical policy indicators across various geographic levels. Below are a few use cases:

### 3.1 Breastfeeding campaign

The district collector of Hardoi District, Uttar Pradesh, aims to improve breastfeeding rates and requires village-level data for effective resource allocation. Leveraging India Policy Insights, officials specify "Villages" as the geographic focus and refine by district. Entering "Breastfed" as a search term retrieves relevant indicators, such as "Initial Breastfeeding." The Explore Data function then generates rankings and visualizations, supporting detailed analysis of breastfeeding rates across villages.

### 3.2 Policy action agenda

The vice chairman of NITI Aayog seeks to refine the national policy action agenda through an analysis of district-level NITI indicators across India. Using India Policy Insights, the vice chairman accesses a nationwide view of districts, selects relevant indicators, and leverages the Explore Data and Compare functionalities to evaluate district data. By highlighting districts on an interactive map and selecting all districts, the tool enables a comprehensive comparison, allowing for the identification of performance gaps and supporting informed policy adjustments.

### 3.3 Officer training on data usage

An instructor at the Centre for Food Planet and Health, LBSNAA, incorporates India Policy Insights into officer training to build competencies in analysing health, nutrition, and population indicators. Officers are guided through the IPI tool by selecting "Districts" as the focus area and using the Deep Dive feature to access detailed metrics. They explore the Health and Nutrition categories by searching for specific indicators or opting to select all indicators, equipping them with the analytical skills essential for policy development.

### 3.4 Government annual report

An office assistant in the Department of Education, Hardoi District, needs population development metrics, such as literacy and graduation rates, for the annual report, including comparisons to state and national averages. The assistant uses India Policy Insights, selecting "Districts" as the geographic scope and accessing metrics within the Education domain through the Deep Dive feature. By selecting specific indicators or opting to include all, the assistant compiles a comprehensive dataset, supporting the preparation of a thorough comparative report on educational performance.

## 4 Impact

The India Policy Insights (IPI) platform has a vast impact across Public Policy, Public Health and Sustainable Development Goals (SDGs). By empowering governments, academics, policymakers, and the public, it provides an effective tool to assess and address local challenges with precision. The platform uses data from key sources and advanced software to provide geo-visualized insights for policy decisions. Its full public accessibility fosters transparency and enables the tracking of developmental progress, driving positive change at various administrative levels. It ensures democratization of geospatial analytics for diverse stakeholders. By bridging the gap between complex datasets and actionable insights, IPI strengthens the foundation for evidence-based governance for the achievement of developmental goals.

In addition to its practical applications, IPI's software design principles and methodologies provide a replicable framework for developers aiming to create similar tools. The modular architecture, which separates backend services, frontend interfaces, and data processing workflows, offers a flexible framework that can be adapted to other geospatial and policy-focused tools. By leveraging containerized deployment via Docker and ensuring compatibility with scalable cloud environments like Azure, IPI demonstrates how to handle large, complex datasets efficiently. The integration of intuitive, user-friendly design principles also provides guidance on building accessible interfaces that cater to users with varying levels of expertise.

## 5 Conclusions

The India Policy Insights (IPI) platform represents a significant advancement in the application of geospatial technology to public policy by bridging the gap between complex datasets and actionable insights. By providing access to 122 population, health, and socioeconomic indicators, the platform helps users address regional disparities accurately and transparently. Built on React, .Net, PostgreSQL, and Azure, IPI's architecture ensures scalability, reproducibility, accessibility, and high performance under varying workloads. The platform's versatile features, including the Deep Dive, Compare, and Atlas tools, facilitate granular analysis at multiple geographic levels, from villages to districts. The reliance on open-source technologies like React, .NET, and PostGIS promotes transparency, collaboration, and the potential for further development and customization.

As a future direction of research, we plan to develop an IPI Data Explorer for village-level data in India, enabling more granular geographic analysis. Although the database structure supports adding any amount of data sets, in case for example, of additional years data, the UI would need to be revised to accommodate the filters/navigation. This could also cause a degradation in the overall performance, new strategies to maintain the database performance could be applied such as table partitioning or scaling the underlying instance. One could also consider the use of a different cloud provider–since all the services are containerized the platform could be run in any provider that supports Docker images and PostgreSQL databases. Additionally, we aim to leverage our backend PostgreSQL databases to enable complex spatial analysis alongside visualization. Finally, we aim to enhance the platform with GenAI-powered features, allowing users to seamlessly transform natural language queries into actionable insights. These enhancements will provide geospatial visualizations and narrative explanations, bridging the gap between complex geospatial analytics and accessibility for policymakers, researchers, and the public.

## CRediT authorship contribution statement

**Devika Jain:** Writing – review & editing, Writing – original draft, Data curation. **Joaquin Kachinovsky:** Writing – review & editing, Writing – original draft, Software, Data curation. **Gonzalo Rodriguez:** Writing – review & editing, Software. **Junyi Chen:** Writing – review & editing, Writing – original draft. **Rockli Kim:** Writing – review & editing, Conceptualization. **S V Subramanian:** Writing – review & editing, Supervision, Conceptualization.

## Declaration of competing interest

The authors declare that they have no known competing financial interests or personal relationships that could have appeared to influence the work reported in this paper.
